# Keyword Index for Volume 88

**DOI:** 10.1038/sj.bjc.6601555

**Published:** 2003-12-09

19q 1914

2-(4-aminophenyl)benzothiazole 470

2-(4-aminophenyl)benzothiazoles 599

5′-deoxyfluorouridine 957

5-fluorouracil 957, 1507

5-FU 624, 1669

5HT_3_ antagonists 1823

5q21 region 1925

7q31 1909

7q31 region 1925

8p21 270

*α*1-adrenoceptor antagonists 1615

*α*5*β*1 integrin 327

*α*-fetoprotein 1878

*α*-particle emitter ^213^Bi 944

A33 antibody 937

acrylamide 84

adaptive intrapatient dose adjustment 814

additivism 1281

adenocarcinoma 63, 1229

adenocarcinoma of the cardia 401

adenoma size 1866

adenoma–carcinoma 1866

adenomatous polyposis coli (APC) 202

adenovirus 1492

adhesion molecules 1773

adrenal gland 1747

advanced non-small-cell lung cancer 342

adverse drug reaction 1157

adverse effects 1510

Africa 1

age 487, 839

agmatine 613

ALA derivatives (ALA hexyl ester) 432

alcohol 1044, 1702

alcohol drinking 58, 366

alcoholism 1044

alkylating agents 1828

alternative splicing 918

AMD473 1128

amelanotic melanoma 1462

amifostine 754

aminolevulinic acid (ALA) 432

anaemia 988, 1851

anal cancer 1352

androgen-independent 822

anger 658

angiogenesis 163, 307, 478, 537, 553, 718, 796, 1424, 1453, 1484, 1727, 1894

angiogenesis inhibitors 965

angiogenic factor 307

Annexin V 1301

antiapoptosis 902

antibiotics 1012

antibody directed enzyme-producing therapy (ADEPT) 937

antibody pretargeting 996

anticancer effect 1948

Antiemetics 1823

antineoplastic agents 1794

antioxidants 940, 1381

antitumoral agents 940

antivascular 1844

anti-VEGF mAb 1979

anxiety 658

Apaf-1 1786

APC 1932

apheresis 396

aponecrosis 1987

apoptosis 123, 161, 163, 210, 223, 298, 487, 586, 748, 754, 788, 910, 918, 1077, 1143, 1301, 1527, 1584, 1615, 1649, 1764, 1786, 1948, 1971

apoptosis induction 1801

areca 366

arginase 613

arginine 613

arginine decarboxylase 613

aromatase 630

aryl hydrocarbon receptor 599

association 1920

atrophic gastritis 1239

augmentation 832

autocrine 567

autophagy 1277

axillary nodes 104

Axin 1735

*β*-catenin 206, 1756

*β*-catenin 740

B7.1 424

B7.2 424

*bak* 1584

Barrett's oesophagus 1271

BAX 586, 848

BCL-2 848

Bcl-X_L_ 910

Belgium 560

benign prostate hyperplasia 928

bereavement 1698

bilateral testicular neoplasms 828

bile acids 748

biochemotherapy 424

biological markers 1721

biomarkers 1239, 1271

bisphosphonates 1631, 1971

black gold miners 1361

bladder cancer 92, 586, 1492, 1932

bleomycin 1764

blood supply 291

blood vessel invasion 1894

BMI 36

BNIP3L 270

body mass 1038

body mass index 1388

body weight 679

bone infiltration 1105

bone marrow 855

bone metastasis 195, 1318

bone resorption 1318

bone scan 195

bone sialoprotein 396

bosentan 788

Bowen's disease 1375

BRAF 1403

brain tumours 1889

BRCA1 1256, 1285

BRCA1-defective cells 1285

BRCA1 protein 1263

BRCA2 933, 1256

breast 362, 702, 1339

breast and prostate cancer 438

breast cancer 50, 58, 133, 277, 406, 470, 491, 574, 630, 832, 863, 871, 944, 1047, 1065, 1077, 1084, 1091, 1168, 1207, 1256, 1310, 1318, 1358, 1400, 1587, 1708, 1894

breast cancer diagnosis 832

breast cancer resistance protein 878

breast cancer risk 1394

breastfeeding 1035

breast hereditary cancer 1285

breast imaging 4

breast implants 832

breast neoplasms 102, 832

breast self-examination 1047

breast tumours 579

bromodeoxyuridine 895

Burkitt's lymphoma 1566

butyrate 748

bystander effect 767

c-*fos* 1143

c-kit 1157

c-*myc* 1143

C4.4a 579

CA-19-9 1248

calcitonin 1537

cancer 163, 223, 270, 684

cancer immunotherapy 1292

cancer registries 1693

cancer susceptibility 487

cancer therapy 613

capecitabine 782

CAR 1411

carbonic anhydrase 1065

carcinogenesis 733, 740, 1217, 1501, 1735

carcinoma 702, 1543

carcinosarcoma 654

cardiotoxicity 1507

carotenoid 1381

case–control 58

case–control studies 373, 1702

caspase-3 1615

caspase-8 1527

caspase-9 1971

caspase-activated DNase 210

caspases 910, 1801

catabolism 613

caveolin-1 1234

CCR2 855

CD24 231

CD26/DPPIV 455

CD34 selection 1874

CD40 1527

CD40L 586

CDH1 1932

CDKN2A 1920

CEA 1248

cell cycle 455, 1417, 1470, 1649

cell cycle arrest 388, 754

cell death 613

cell death assays 125

cell invasion 1111

cell nucleolus 1942

cell proliferation 775

cervical cancer 1054, 1095, 1213, 1388, 1584

cervical cancer screening 560, 1570

cervical carcinoma 63

cervical screening 42

cervix cancer 1713

CGH 1578

chemokines 855, 1773

chemoradiation 1352

chemoradiotherapy 17

chemoresistance 624

chemotherapy 25, 167, 181, 187, 491, 496, 654, 1025, 1084, 1180, 1185, 1199, 1248, 1352

Child Health Questionnaire 1185

childhood cancer 373, 382, 1035, 1661

childhood leukaemia 775, 1939

childhood solid tumours 1370

children 1693

chimeric 1119

chimeric receptor 1292

CHO-K1 327

cholecystectomy 79

chromogranin 1747

chromosome 9 1578

chromosome aberrations 548, 1939

chronic disease 1851

chronic lymphatic leukaemia 74

chronic lymphocytic leukaemia 593

circumferential resection margin 1017

cirrhosis 733

CISH 1587

cisplatin 33, 814, 951, 1199, 1285, 1516

cisplatin-resistance 1327

citrulline 613

clear cell ovarian 1578

clinical trials 1128, 1661

CML 983

cohort study 684

collagen crosslinks 1105

collateral sensitivity 1963

collusion 839

colon 895

colon adenoma cancer 748

colon adenomas 79

colon cancer 79, 530, 788, 1445, 1756

colon carcinoma 937, 1995

colorectal 206

colorectal cancers 648, 1038, 1044, 1598, 1859, 1909

colorectal carcinoma 413, 726

combination chemotherapy 1942

comet assay 895

communication 666

communication skills 502, 658

comparative genomic hybridisation 1914

confocal microscopy 146

cost-effectiveness 31

costimulatory molecules 1527

coumarin derivatives 1111

COX-2 574, 1217, 1631

COX inhibitors 1143

CpG island 117, 413

CpG methylation 521

CPG2 1622

CPT-11 1979

crossreactivity 202

CXCR-4 1631

cyclin 1417

cyclin B1 257

cyclin D1 257

cyclin D2 1560

cyclin E 1914, 1956

cyclin-dependent kinase inhibitors 1277

cyclooxygenase-2 1445, 1598

cyclosporin 973

CYFRA 21-1 1894

CYP17 933

CYP1A1 599

CYP3A4 928

cystine 951

cytodiagnosis 1741

cytokines 630, 1207, 1773

cytological techniques 1883

cytology 702

cytology performance 1570

cytoprotection 754

cytoskeleton 1794

cytotoxic medium conditioning 447

Dacarbazine 132, 496

darbepoetin alpha 1851

decision making 1675

delayed reproductive death 548

deletion spectrum 90

demethylation 1560

denaturing HPLC 1889

*de novo* carcinoma 1866

deoxycytidine kinase 1963

depression 658

deprivation 613

detection 1358

DFF 210

DFF-B 210

DHPLC 270

diagnosis 354, 516, 839, 1175, 1239, 1358, 1878

diet 84, 1381, 1682

DMXAA 1160, 1844

DNA adducts 470

DNA damage 599

DNA fragmentation factor 210

DNA methylation 1932

DNA-ploidy 31

DNA repair 1939

DNA replication 895

DNA sequence 1403

docetaxel 11, 1168, 1335, 1339, 1669

documentation 839

dorsal root ganglion 1942

doxorubicin 1281, 1285, 1956

DPC4 420

drug resistance 775, 951, 973, 983, 1794, 1956

dwelling time 1866

dysadherin 726

dysphagia 18

dystroglycan 579

E-cadherin 1727

E-cadherin 718

*E. tirucalli* 1566

E1B-deleted 1492

early breast cancer 104

ECM 1470

EGF binding 1327

*Egr-1* 1143

electrochemotherapy 1764

electromagnetic fields 1939

electroporation 1764

endocrine tumours 1747

endogenous hormones 1394

endometrial cancer 1175

endometrial carcinoma 245

endometrial thickness 1175

endosomal/lysosomal acidification 1327

endothelial cells 307, 1484

endothelin 788

endothelin-1 163

Ep-CAM 574

epidemiology 63, 74, 382, 679, 684, 1032, 1370, 1682, 1687, 1698

epidermal growth factor 796

epithelial cancers 1501

epithelial ovarian carcinoma 237

Epstein–Barr virus 1566

ErbB2 396

erythropoietin 988, 1851

ethnicity 277

Etoposide 25, 140

Ewing's sarcoma 137

EWS 145

exercise 679

explant 767

extracellular matrix 871, 1248

extrahepatic bile duct carcinoma 1234

factors 988

familial 1256

Fas 1301

fascin 537

FasLigand 788

febrile neutropenia 181

FGF8b 1432

FHIT 1501

Fhit expression 1213

fibrin 718

fibulin 871

fine-needle aspiration 354, 699, 702

FISH 1432, 1587

Flanders 560

Fli-1 137

fluid-based specimen 1883

fluid-phase/receptor-mediated endocytosis 1327

fluoropyrimidines 648

fluorouracil 1017, 1510, 1859

folinic acid; adjuvant therapy 1859

food additives 940

formulation 50

Foscan® 146, 283

fotemustine 496

fragile sites 1501

fruit 1388

ftorafur 957

functional screening 910

fusion proteins 1622

*γ*-radiation 388

G1/S transition 1417

G2 arrest 1470

G_2_–M 455

gallstones 79

gastric cancer 796, 1909, 1914

gastric carcinoma 1560

gastrin-releasing peptide 1809

gastrointestinal tract 1747

gelsolin 606

gemcitabine 491, 1963

gene amplification 1587

gene expression profiles 1058

gene–gene interaction 928

gene therapy 1492

genetic counselling 1675

genetic markers 1741

genomic instability 548

genotoxic agents 298

glioblastoma multiforme 496, 516

glioma 463, 496, 1277, 1411, 1439

glutathione 951

Goseki classification 401

GPCR 567

growth factor 1522

GSK-3*β* 1470

GST genotypes 66

guidelines 1191

haem oxygenase 902

haematogenous dissemination 1894

haemodialysis 25

hAG-2 579

hAG-3 579

head and neck cancer 1012, 1217

head and neck carcinoma 1741

health-related quality of life 1185

health services 1025

Health Utilities Index 1185

height 1038

HeLa 613

*HELG* 1909

*Helicobacter pylori* 1239

hepatic stellate cells 733

hepatitis viral infection 521

hepatoblastoma 373

hepatocellular carcinoma 210, 521, 1878, 1909

hepatocytes 733

hepatoma 973

hepatoma-specific band of serum gamma-glutamyl transferase 1878

HER2 1032, 1587

HER-2/neu 1292

hereditary tumours 1285

hierarchical clustering 510

high-dose chemotherapy 1831, 1874

high-grade osteosarcoma 1925

hip implants 548

histidine triad 1501

histological type 1866

histology 843

HIT 1501

HMGI-C 1406

*hMLH1* 521

Hodgkin's disease 1335

Hoechst 33342 1979

homing 855

hormone factor risk 1032

hormone responsiveness 1071

HPV 251, 1213

HPV 16 serology 1095

HPV test performance 1570

HPV testing 42

*hTERT* 516

human 1721

human Mdm2 636

human neuroblastoma 1527

human papillomavirus (HPV) 63, 560, 1713, 1883

human sterol isomerase 438

Hybrid Capture II 1883

hyperammonia 447

hypermethylation 109, 1560

hyperthermia 1839

hypoxia 120, 307, 718, 1065, 1439

IAPs 1077

IDN 5390 965

ifosfamide 1168

IGF1 277

IGF-I 1682

IGFBP-3 1682

I*κ*B*α* 624, 1615

I*κ*B kinase-*α* 1598

IL-10 1605

IL-12 1453

IL-2 320

ILK 1470, 1756

image analysis 1439

image processing 1453

imatinib 983, 1157

immunocytochemistry 537

immunoglobulin gene 593

immunohistochemistry 231, 726, 1077, 1223, 1234, 1263, 1587, 1598, 1727, 1735

immunoreactivity 1432

immunostaining 257

immunotherapy 175, 348, 1135, 1346, 1641

*in situ* carcinoma 1375

*in situ* DNA synthesis 257

*in vivo* electropermeabilization 1764

independent prognostic factor 1406

induction 11, 1339

inflammation 1207, 1773

inflammatory breast cancer 718

iNOS 120

insulin 263

insulin-like growth factor-1 277

insulin-like growth factor-2 733

interleukin 6 630

interleukin-12 1641

interleukin-2 1641

interstitium 291

intracellular localisation 146

intrahepatic cholangiocarcinoma 1894

intrapleural therapy 1839

invasion 463, 1631

invasiveness factors 1207

ionising radiation 1786, 1801

irinotecan 335, 1180, 1516

ischaemia-reperfusion injury 760

isolated hepatic perfusion 314

Jak-STAT1 signalling 1995

Japanese 1038

Jurkat 455

Kaposi's sarcoma 1

kidney 327

kidney cancer 84

KOC 699

KRAB 137

kringles 1–5 1492

KSHV/HHV-8 1

Ku80 202

L1210 613

lactacystin 120

LAR 1223

large bowel cancer 84

lauryl gallate 940

LDB1 1543

leiomyosarcoma 510

leptomycin B 636

LIM 1543

limited proteolysis 636

linkage analysis 1920

lip cancer 1702

lipid droplets 1439

literature review 63

liver metastases 314

LMO4 1543

LOH 251, 270, 1578

long-term survival 496, 1666, 1894

loss of heterozygosity (LOH) 420, 1889

low-dose aspirin 684

lung cancer 606, 808, 1025, 1229, 1727

lung resistance-related protein 878

lymph node 553, 702

lymph node metastasis 237

lymphadenopathy 354

lymphodepletion 1874

lymphoma 140, 553

lymphopenia 181

macromolecule uptake 291

macrophage inhibitory factor-1 1101

magnetic resonance imaging 1017, 1135

magnetic resonance mammography 4

male and female breast cancer 933

malignancy 354

malignant fibrous histiocytoma 510

malignant melanoma 74, 175, 1358

malignant mesothelioma 1553

malignant neoplasms 362

malignant pleural mesothelioma 167

mammary carcinoma 1995

mammography 362

mannose 6-phosphate/insulin-like growth factor-2 receptor 733

MAP kinase 748

marker 1065, 1191

marker genes 1091

Markov model 1866

*maspin* gene expression 863

mast cells 1157

matrix metalloproteinases 1318, 1445, 1553

MCF-7 567, 599, 1310

MDA-MB-231 cell line 944

medullary thyroid cancer 1537

MEK 848

melanoma 132, 291, 424, 1157, 1381, 1403, 1920

melphalan 314

memory T cells 223

mesothelioma 1839

mesothelioma cells 388

meta-analysis 1047, 1191

metabolism 782

metastases 702

metastasis 96, 537

metastatic breast cancer 1406, 1631, 1669

metastatic colorectal cancer 1248

metastatic disease 491

methylation 217, 413, 463

MGMT 521

MHC II 424

microarray 510, 1995

microbeam 767

microcirculation 1462

microdialysis 782

microsatellite instability 413

microsatellite repeats 1741

microsatellites 1925

microspectrofluorometry 154

microtubules 1794

microvasculature 291

microvessel density (MVD) 314, 1424, 1900

midkine 1522

migration 1484

minichromosome maintenance (Mcm) proteins 257

mistletoe lectin 1786

mitogenesis 567

mitomycin 1516

mitosis 1649

mitotic cell death 1764

mitoxantrone 491

MMP-2 1248

molecular target 1522

monoclonal antibody 996

monocytes 1207

mortality 1047

mouse fibrosarcoma 760

MRI 478, 1592

mRNA 206

MtDNA 90

mucositis 1012

multicentre phase II study 1346

multidrug resistance 878, 1963

multidrug resistance-associated protein 1 878

multiple intestinal neoplasia 1445

multiple myeloma 855

multiple skin cancers 1375

multivariate analysis 1721

mutant p21 530

mutations 109, 206, 1271, 1403, 1584, 1909

N-cadherin 1727

N15 antibody 202

naïve T cells 223

napsin 1229

nasopharyngeal carcinoma 187

negative screening 1054

neoadjuvant 187, 1339

neoadjuvant chemotherapy 406, 1017

neoplasms 988, 1358

neoplastic cells 918

neovascularization 110

nervous system tumours 109

NESP55 1747

neuroblastoma 478, 1522, 1641, 1874

neurokinine receptor antagonists 1823

neuropilin 796

neurotoxicity 1942

nevus density 1920

NF-*κ*B 120, 153, 624

nitric oxide 902, 1484

nitroimidazole 307

NK_1_ antagonists 1823

non-Hodgkin's lymphoma 74, 1335

nonplatinum regimen 342

non-small-cell lung cancer 231, 335, 537, 814, 1666

nonviral vectors 1641

normal prostate NPTX 1532 cells 1605

Northern Ireland 1256

NSAIDs 1687

nuclear factor-*κ*B 1598

nuclear localisation 115

ODC 1143

oesophageal cancer 1549

oesophageal carcinoma 18, 1095

oesophageal squamous cell cancer 115

oesophageal squamous cell carcinoma 1217, 1735

oestrogen 50

oestrogen receptor 579, 871, 1071

oncological interviews 502

oral 648

oral contraceptives 50, 1713

oral leukoplakia 366

oral route 965

oral squamous cell carcinoma 1105

oral submucous fibrosis 366

ornithine 613

ornithine cycle 447

oropharyngeal carcinoma 1095

osteoclast 1318

osteosarcoma 396, 1995

ovarian cancer 470, 654, 666, 839, 848, 1781

ovary 1828

oxaliplatin 1942

oxidative stress 902

oxygen 1462

P-glycoprotein 878

P-subunit 1223

p14 217

p16 217

p21 protein 754

p21^ras^ 1971

p27^KIP1^ 1277

p53 251, 298, 636, 1281, 1310, 1492, 1649, 1786, 1925

p53 gene 1271, 1741

p53 protein 754

p63 740

paan chewing 1388

paclitaxel 1285, 1649, 1942

paediatric population 1925

PAI-1 96

pancreas 1747

pancreatic cancer 530, 679, 1180, 1971

pancreatic carcinoma 217

pancreatitis 217

parental smoking 373

pathology 654

patient 1119

patient information 1661

patient–physician relationship 658

pediatric 1185

penile carcinoma 1095

pepsinogen 1239

peptide vaccination 530

perfusion 291, 1439, 1979

perilesional skin 90

peripheral blood progenitor cell transplantation 1831

peritoneal cytology 245

PF regimens 11

pharmacodynamics 1160

pharmacokinetics 283, 782, 814, 1160, 1942

phase I 1128

phase I clinical trial 1844

phase I/II study 491

phase I study 1168

phase II 648

phenylacetate carboxymethyl benzylamide dextran (NaPaC) 1987

Phortress 470

phosphohistone H3 257

phosphoinositides 606

photodynamic diagnosis 1781

photodynamic therapy 283, 1773

photodynamic therapy (PDT) 432, 760

physical activity 679

physicians’ locus of control 502

PKC 606, 748

plasminogen 1492

plasminogen activation inhibitor type 2 944

platinum 1128

platinum DNA adducts 814

pleural malignancies 1839

PMN-elastase 1084

polymerase chain reaction 1883

polymorphism 263, 928

population-based cohort study 382

population mixing 1370

prediction 1091

predictive markers 406

predisposing 988

prevention 1687

primary cells 1071

primary liver carcinoma 1894

progestin 50

prognosis 18, 102, 237, 348, 516, 537, 574, 593, 726, 1065, 1191, 1549, 1553

prognostic factors 1199, 1510, 1537

prognostic indicator 1077

prognostic markers 31, 115, 586

prognostic value 401, 863

prolactin 1301

prolactin receptor 1301

proliferation 613, 767, 1143, 1484

proliferation inhibition 438

prophylactic mastectomy 1675

prophylaxis 1507

prospective study 1394

prostaglandin E_2_ 630

prostate cancer 31, 195, 263, 822, 928, 1101, 1358, 1432, 1615, 1682

proteasome 636

protein 1417

protein induced by vitamin K absence or antagonist II 1878

protein kinase C alpha 1400

protein p53 1794

protein tyrosine kinase inhibitors 940

protein-tyrosine kinase 1058

proteolysis 871

protoporphyrin IX 1462

protracted intravenous infusion 1859

*Pseudomonas* exotoxin 1327

psychological stress 1698

psychosocial impact 42

PTHRP 567

PTP 1223

pyridinoline 1105

quality of life 988

quantitative RT–PCR 1101

quinazolines 1615

radiation 487

radiosensitivity 128, 1470

radiotherapy 187, 828, 1012, 1017, 1025, 1352, 1584

raltitrexed 1180

randomised phase III trial 335

rat fibroblasts 1956

*RAY1* 1909

Rb 1925

*RB1* gene 109

real-time PCR 516, 1091

real-time RT–PCR quantification 863

receptor 1119

recombinant 988

recombinant fusion proteins 937

records 839

relapse-free survival 1537

relative risk 1054

renal-cell carcinoma (RCC) 348, 1346, 1417, 1516, 1801

renal insufficiency 25

repressor 137

resection margin 1549

residual neoplasms 843

response 424

response biomarker 1592

response to therapy 1084

retinoic acid 1058

retinol 1381

retrovirus 1119

RhoA 1631

risk 348, 684, 1687

risk factors 79, 181, 366, 1702

risk model 181

risk perception 1675

ruthenium(III) 1484

S-1 648

S-phase arrest 599

Sant 7 630

Scotland 1256

screening 1708

SDF-1 1631

seasonal variation 1358

second line 1828

second-line treatment 1180

self-efficacy 658

senescence, apoptosis 463

sequential-triplet chemotherapy 342

sex behaviour 666

sex ratio in offspring 382

sigma receptors 438

signal transduction 796, 1281

signal transduction pathways 1801

signalling 153

silicone 832

single nucleotide polymorphism 1735

skin cancer 98

Smad 1615

Smad4 420

smoking 366

soft tissue sarcoma 510

solid tumours 1831

soluble CD105 1424

somatic hypermutation 593

somatostatin 140

South Africa 1361

SP-G 1809

specialisation 1708

spectroscopy 478

spermatogonia 828

spheroid model 463

sporadic breast cancer 1263

squamous cell carcinoma 63

squamous cell carcinoma of the head and neck 11

SR31747A 438

*ST7* 1909

staging 1105

standardised incidence ratio 1666

standardised mortality ratio 1666

statistical methods 1693

statistical models 843

stratification 348

substance *P* 1823

substance P analogues 1809

sun exposure 1702

surfactant protein 1229

surgery 1025, 1549

survival 348, 1025, 1693, 1698, 1708

survival-analysis 102, 1191

survival rate 1721

survivin 1077

survivin expression 115

synovial sarcoma 510

synthesis 1948

systematic review 1191

T-cell homeostatic proliferation 1874

T-cells 530

T lymphocyte 1119

tamoxifen 1084, 1175

tamoxifen resistance 1400

tandem duplications 90

targeted *α* therapy 944

targeting 1622

taxanes 965, 1339

Taxol 973

TCF 1932

telomere length 593

temozolomide 175

tenascin 996

testicular biopsy 828

testicular cancer 36

testicular germ-cell tumours 878

testicular intraepithelial neoplasia 828

testicular neoplasms 828

testis 843

TGF-*β*1 1615

TGF*β* 420, 1424

thalidomide 822

therapy 478

thymidine kinase 1963

thymidine phosphorylase 957

thymidine phosphorylase inhibitor 957

thyroid cancer 1358

thyroid carcinoma 1223

TIL 320

time course 1510

time-lapse microscopy 1310

TIMP-1 1248, 1605

tissue exposure 782

tissue microarray 231, 1417

TNF-*α* 314

tobacco 1702

tocopherol 1381

tomudex 624

topoisomerase I 1310

topoisomerase I inhibitor 808

topoisomerase II *α* 455

topoisomerase II inhibitor 808

topotecan 1310

toxicity 181, 1160, 1199, 1831

TP53 848

TRAIL 153, 298, 910, 918, 1801

TRAIL receptors 298

transport mechanisms 291

transporter 951

transvaginal ultrasonography 1175

treatment 36

trifluorothymidine 957

TT-232 140

TTF-1 1229

tumorigenesis 420

tumour angiogenesis 1987

tumour delivery 1979

tumour growth 163, 902, 1111, 1809

tumour immunosuppression 320

tumour marker 396, 699, 1522, 1894

tumour metastasis 327

tumour perfusion 1592

tumour response 406

tumour suppression 606

tumour suppressor 1411, 1501

tumour-suppressor gene 1909

tumour targeting 937

tumours 1119, 1135, 1462

tyrosine phosphorylation 1605

ultrasound 354, 702

uPA 96

urea/bisulphite sequencing 521

urinary sex steroids 1394

urothelium 740, 767

vaginal and vulvar cancer 1095

validity 843

vascular damage 283

vascular endothelial growth factor (VEGF) 796, 1987

vascular phenotype 553

vascular regression 553

vascular targeting agents 1592

vegetables 1388

VEGF 1622

VEGF-C 237

VEGF-D 237

VEGFR-3 237, 1453

VIN 251

vinorelbine 1281

viral gene expression 1566

visible reflectance spectroscopy 760

vitamin D receptor 928

vitamin E analogues 1948

vitamin E succinate 153

vulval cancer 251

vulval intraepithelial neoplasia (VIN) 257

warts 1702

weekly chemotherapy 808

women 1381

X-rays 447

xanthine oxidase 760

xenograft 478

*ζ* chain 223

ZD6126 1592

zoledronate 1971

zoledronic acid 1971

zometa 1971

